# Subgroup and Prognostic Factor Analysis in T4 Lung Cancer Based on the 9th Tumour-Node-Metastasis Classification

**DOI:** 10.1093/icvts/ivaf276

**Published:** 2025-11-23

**Authors:** Bekir Elma, Ahmet Uluşan, Maruf Şanlı, Ahmet Ferudun Işık

**Affiliations:** Department of Thoracic Surgery, Faculty of Medicine, Gaziantep University, 27310 Gaziantep, Turkey; Department of Thoracic Surgery, Faculty of Medicine, Gaziantep University, 27310 Gaziantep, Turkey; Department of Thoracic Surgery, Faculty of Medicine, Gaziantep University, 27310 Gaziantep, Turkey; Department of Thoracic Surgery, Faculty of Medicine, Gaziantep University, 27310 Gaziantep, Turkey

**Keywords:** T4 non-small-cell lung cancer, TNM 9th edition, surgical outcomes, nodal staging, adjuvant therapy

## Abstract

**Objectıves:**

T4 non-small-cell lung cancer comprises a biologically and anatomically heterogeneous group. The 9th edition of the Tumour-Node-Metastasis staging system introduced refined T4 definitions and subdivided N2 disease into single- and multi-station involvement. This study aimed to assess long-term survival and prognostic factors in surgically treated T4 non-small-cell lung cancer patients, focusing on T4 subgroups and nodal status.

**Methods:**

We retrospectively analysed patients who underwent resection for pathologically confirmed T4 non-small cell lung cancer between 2006 and 2024. Patients were categorized based on T4 criteria: tumour diameter >7 cm, adjacent structure invasion, or multiple T4 features. Survival outcomes were assessed using Kaplan-Meier, Aalen-Johansen and Cox regression analyses.

**Results:**

A total of 191 patients were analysed. The 5-year overall survival rate was 34.1%, varying across subgroups: 38.7% (size), 29.9% (invasion), and 6.1% (multiple criteria) (*P* < .001). Adjuvant chemotherapy was associated with improved overall survival (hazard ratio [HR]: 0.511; *P* = .001), while N2 disease (HR: 1.750; *P* = .012) and multiple T4 features (HR: 2.590; *P* < .001) predicted worse outcomes. Similar patterns were observed in recurrence-free survival. N2 involvement was particularly adverse in the invasion group.

**Conclusıons:**

T4 aetiology and nodal status significantly impact survival following surgery. These findings support the prognostic utility of the 9th Tumour-Node-Metastasis edition and underscore the value of tailored surgical strategies.

## Graphical Abstract

**Figure:**
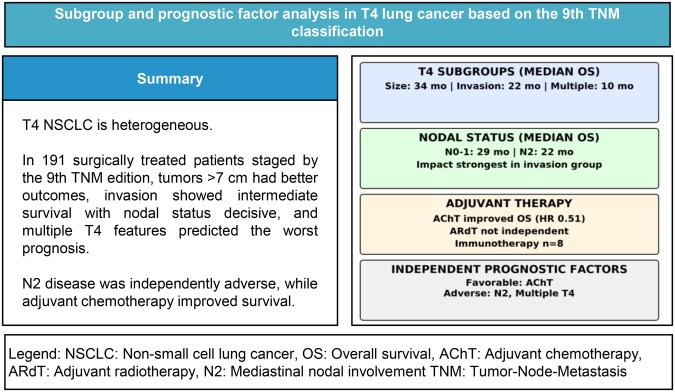

## INTRODUCTION

Prognostic factors in lung cancer critically influence tumour behaviour, treatment response, and survival. Parameters such as age, sex, histology, tumour diameter, nodal involvement, metastasis, and treatment type help guide personalized therapeutic strategies.^1^

In the 8th edition of the Tumour-Node-Metastasis (TNM) staging system, tumours >7 cm were classified as T4 for the first time.^2^ The 9th edition retained the core T descriptors but introduced refined definitions of local invasion, including specific structures such as the diaphragm, great vessels, and vertebrae. Notably, N2 disease was subdivided into single- and multi-station involvement (N2a/N2b), acknowledging the prognostic variability within this category.^3^

T4 non-small-cell lung cancer (NSCLC) encompasses a biologically and anatomically heterogeneous group. While some T4 tumours result from large diameter alone, others involve extensive invasion into adjacent structures or multiple criteria, with varied implications for resectability and prognosis. Surgical resection in selected T4 cases has shown acceptable outcomes, particularly with complete resection (R0) and limited nodal disease.^4–6^

Despite advances, data on how different T4 features and nodal statuses affect long-term survival remain limited. This study evaluated survival outcomes and prognostic indicators in a large cohort of surgically treated T4 NSCLC patients, emphasizing T4 subclassifications and nodal stratification based on the 9th edition TNM system.

## MATERIALS AND METHODS

### Ethical approval

This study was approved by the Clinical Research Ethics Committee of Gaziantep University (21/05/2025; No. 2025/104) and conducted in accordance with the Declaration of Helsinki. Data were collected and stored in compliance with institutional and WMA Declaration of Taipei guidelines. The institutional ethics committee approved and oversaw the database’s establishment and use. All patient data were anonymized before analysis, and no identifiable information was used.

### Study design and patient selection

All patients underwent preoperative ^18^F-fluorodeoxyglucose-positron emission tomography/computed tomography (PET/CT). Those with distant metastases or multiple N2 involvement were excluded. Patients with mediastinal nodes showing SUV <2.5 were considered N2-negative and underwent direct resection.^6^ Single-station N2 cases were evaluated with endobronchial ultrasound-transbronchial needle aspiration or mediastinoscopy, and if confirmed (N2a), proceeded to surgery. Thus, only N0, N1, or N2a patients were eligible.

We retrospectively reviewed NSCLC patients who underwent anatomic resection with pathological T4 between 2006 and 2024, staged per the 9th TNM edition.

Inclusion: no neoadjuvant therapy, R0 resection, complete lymph node dissection, or postoperative detection of unexpected multi-station N2.

Exclusion: neoadjuvant therapy, incomplete anatomic resection or dissection (eg, salvage surgery), R1-R2 resections, and second primary malignancies detected during follow-up or final pathology.

### Preoperative evaluation

All patients underwent pulmonary function testing, cardiac assessment, thoracic CT, PET/CT, and contrast-enhanced brain MRI. Cases were reviewed in a multidisciplinary tumour board.

### Surgical management

All patients underwent anatomical lung resection with systematic mediastinal lymph-node dissection, following ESTS guidelines.^7^ Lobectomy or pneumonectomy was performed for non-invasive tumours, with wedge resection or pneumotomy for ipsilateral nodules. Invasive tumours required procedures such as vascular or pericardial resection, aortic adventitia excision, vena cava graft reconstruction or extended resections involving carina, oesophagus, diaphragm or vertebra.

### Adjuvant therapy and follow-up

Postoperative mortality was defined as death within 30 days. Of 159 patients advised adjuvant chemotherapy (AChT), 139 completed therapy; 32 N2 patients received mediastinal radiotherapy. Only eight patients received adjuvant immunotherapy, which is too few for analysis. Follow-up consisted of CT every 3-6 months, then annually, with biopsy if recurrence was suspected.

### T4 subgroup classification

Patients were categorized as follows:

- Mass size group: patients with T4 due to tumour diameter >7 cm (no other T4 criteria)
- Invasion group: patients with a tumour diameter ≤7 cm and a T4 due to invasion of adjacent structures only, or the presence of a nodule in a different lobe only
- Multiple group: Tumours fulfilling more than one T4 criterion (eg, 7 cm plus invasion).

Although tumour diameter >7 cm defines the “size group,” tumour diameter also overlaps with the multiple T4 group.

All invasion sites were considered under a single “invasion” category to maintain statistical power and avoid overfitting.

### Data collection and survival definitions

Demographics, imaging, pathology, operative notes, and follow-up data were collected. Overall survival (OS) was defined as the time from surgery to death; recurrence-free survival (RFS) was from surgery to recurrence. Recurrence included loco-regional and distant events, whereas patients with second primary malignancies were excluded from the analysis. RFS was chosen in line with recent recommendations favouring this end-point after resection.^8^

### Statistical analysis

Descriptive data were presented as means (SD) or median (IQR) for continuous variables, frequencies for categorical variables. Normality of continuous variables assessed with Shapiro-Wilk test. OS was analysed using the Kaplan-Meier method, as death was the primary event of interest and no competing risks were present for this end-point. In contrast, RFS was analysed using the Aalen-Johansen method, which accounts for death as a competing risk. The log-rank test was used to compare survival over the entire follow-up period; it does not evaluate differences at fixed time points (eg, 5-year survival). Since restricted mean survival time (RMST) analysis requires complete follow-up data and is more appropriate for fixed-time comparisons, it was not applied due to missing data and the retrospective design. Univariable Cox regression identified survival-associated variables, followed by multivariable analysis. Group comparisons used the analysis of variance test, the Kruskal-Wallis test, or the chi-square test, with Dunn’s test for post hoc comparisons. Analyses were performed using R software. A *P*-value <.05 was considered statistically significant.

## RESULTS

### Number of patients

The study included 191 patients; survival analysis was performed on 190 due to missing follow-up in one case (immigrant patient). Median follow-up was 31 months (range, 4-200). By subgroup, median follow-up was 32 months (4-200) for the mass-size group, 30 months (4-178) for invasion and 19 months (7-117) for multiple criteria. Patients were classified as T4 by size >7 cm (*n* = 119), invasion (*n* = 48) or multiple criteria (*n* = 24). Detailed distributions are provided in **Tables 1 and 2**.

**Table 1.** The Invasion Group (Tumours <7 cm): Invaded Structures

Invasive structures	Number of patients, *n* (%)
Pulmonary artery	14 (29.2)
Vertebra	10 (20.8)
Pulmonary vein	9 (18.8)
Trachea	6 (12.5)
Nodule in an ipsilateral different lobe	3 (6.3)
Carina	2 (4.2)
Oesophagus	1 (2.1)
Mediastinum	1 (2.1)
Superior vena cava	1 (2.1)
Aorta	1 (2.1)
Total	48

Distribution of invaded structures in the invasion group (<7 cm). Values are presented as *n* (%).

**Table 2.** Combinations in Multiple T4 Group

Combinations	Number of patients, *n* (%)
Mass size + pulmonary artery	7 (29.2)
Mass size + nodule in an ipsilateral different lobe	4 (16.7)
Mass size + oesophagus	2 (8.3)
Mass size + trachea	1 (4.2)
Mass size + atrium + vertebra	1 (4.2)
Oesophagus + diaphragm	1 (4.2)
Pulmonary artery + pulmonary vein	1 (4.2)
Pulmonary vein + vertebra	1 (4.2)
Pulmonary vein + superior vena cava	1 (4.2)
Inferior vena cava + pulmonary artery + nodule in an ipsilateral different lobe	1 (4.2)
Superior vena cava + pulmonary artery	1 (4.2)
Superior vena cava + vertebra	1 (4.2)
Vertebra + diaphragm	1 (4.2)
Diaphragma + aorta	1 (4.2)
Total	24

A combination of features is shown, each individually meeting the T4 criterion. Values are presented as *n* (%).

### Baseline characteristics and T4 subgroup comparison

The cohort (mean age 62.6 [8.6] years; 94.8% male) included 107 pneumonectomies (56%) and 84 lobectomies (44%). AChT was given to 72.8% and adjuvant radiotherapy (ARdT) to 16.8%. Tumour diameter is shown separately in **Table 3** due to frequent overlap with the multiple group. Complications occurred in 24.6% and reoperations in 9.9%, mainly for bronchopleural fistula (10 of 19 cases). Other clinical and pathological features showed no significant group differences (**Table 3**).

**Table 3.** Baseline Clinicopathological Features by T4 Subgroup

	Total (*n* = 191)	Mass size group (*n* = 119)	Invasion group (*n* = 48)	Multiple group (*n* = 24)	*P* values
Age, mean (SD)	62.6 (8.6)	62.4 (8.8)	61.5 (8.7)	65.7 (6.9)	.122
Gender, *n* (%)					.538
Male	181 (94.8)	114 (95.8)	44 (91.7)	23 (95.8)	
Female	10 (5.2)	5 (4.2)	4 (8.3)	1 (4.2)	
Endobronchial tumour, *n* (%)					.134
Yes	99 (51.8)	55 (46.2)	29 (60.4)	15 (62.5)	
No	92 (48.2)	64 (53.8)	19 (39.6)	9 (37.5)	
Operation type, *n* (%)					.435
Lobectomy	84 (44)	56 (47.1)	20 (41.7)	8 (33.3)	
Pneumonectomy	107 (56)	63 (52.9)	28 (58.3)	16 (66.7)	
Histopathology, *n* (%)					.731
Squamous cell carcinoma	114 (59.7)	70 (58.8)	29 (60.4)	15 (62.5)	
Adenocarcinoma	41 (21.5)	29 (24.4)	8 (16.7)	4 (16.7)	
Other	36 (18.8)	20 (16.8)	11 (22.9)	5 (20.8)	
Tumour diameter (cm) median (IQR)	8 (3)	9 (3)^a^	4.5 (2.5)^c^	7 (3)^b^	.001
pN (9th staging), *n* (%)					.113
N0	85 (44.5)	58 (48.7)	(31.2)	12 (50.0)	
N1	74 (38.7)	45 (37.8)	20 (41.7)	9 (37.5)	
N2a	26 (13.6)	11 (9.3)	(25)	3 (12.5)	
N2b	6 (3.2)	5 (4.2)	(2.1)	0 (0)	
Stage (9th staging), *n* (%)					.086
IIIA	159 (83.2)	103 (86.6)	(72.9)	21 (87.5)	
IIIB	32 (16.8)	16 (13.4)	13 (27.1)	3 (12.5)	
Complication, *n* (%)					.542
No	144 (75.4)	92 (77.3)	(75)	16 (66.7)	
Yes	47 (24.6)	27 (22.7)	12 (25)	8 (33.3)	
Reoperation, *n* (%)					.959
No	172 (90.1)	107 (89.9)	(89.6)	22 (91.7)	
Yes	19 (9.9)	12 (10.1)	5 (10.4)	2 (8.3)	
Adjuvant chemotherapy, *n* (%)					.547
No	52 (27.2)	30 (25.2)	(33.3)	6 (25)	
Yes	139 (72.8)	89 (74.8)	32 (66.7)	18 (75)	
Adjuvant radiotherapy, *n* (%)					.086
No	159 (83.2)	103 (86.6)	(72.9)	21 (87.5)	
Yes	32 (16.8)	16 (13.4)	13 (27.1)	3 (12.5)	

Baseline characteristics of the overall cohort and T4 subgroups. Continuous variables are summarized as mean (SD) or median (IQR) according to distribution; categorical variables as *n* (%). Group comparisons were performed using Kruskal-Wallis or chi-square tests; significant pairwise differences were identified by post hoc Dunn testing (*P* < .05). Superscripts denote significant pairwise differences: ^a^mass size group vs invasion group; ^b^mass size group vs multiple group; ^c^invasion group vs multiple group.

### OS by T4 subgroup

**Figure 1.**
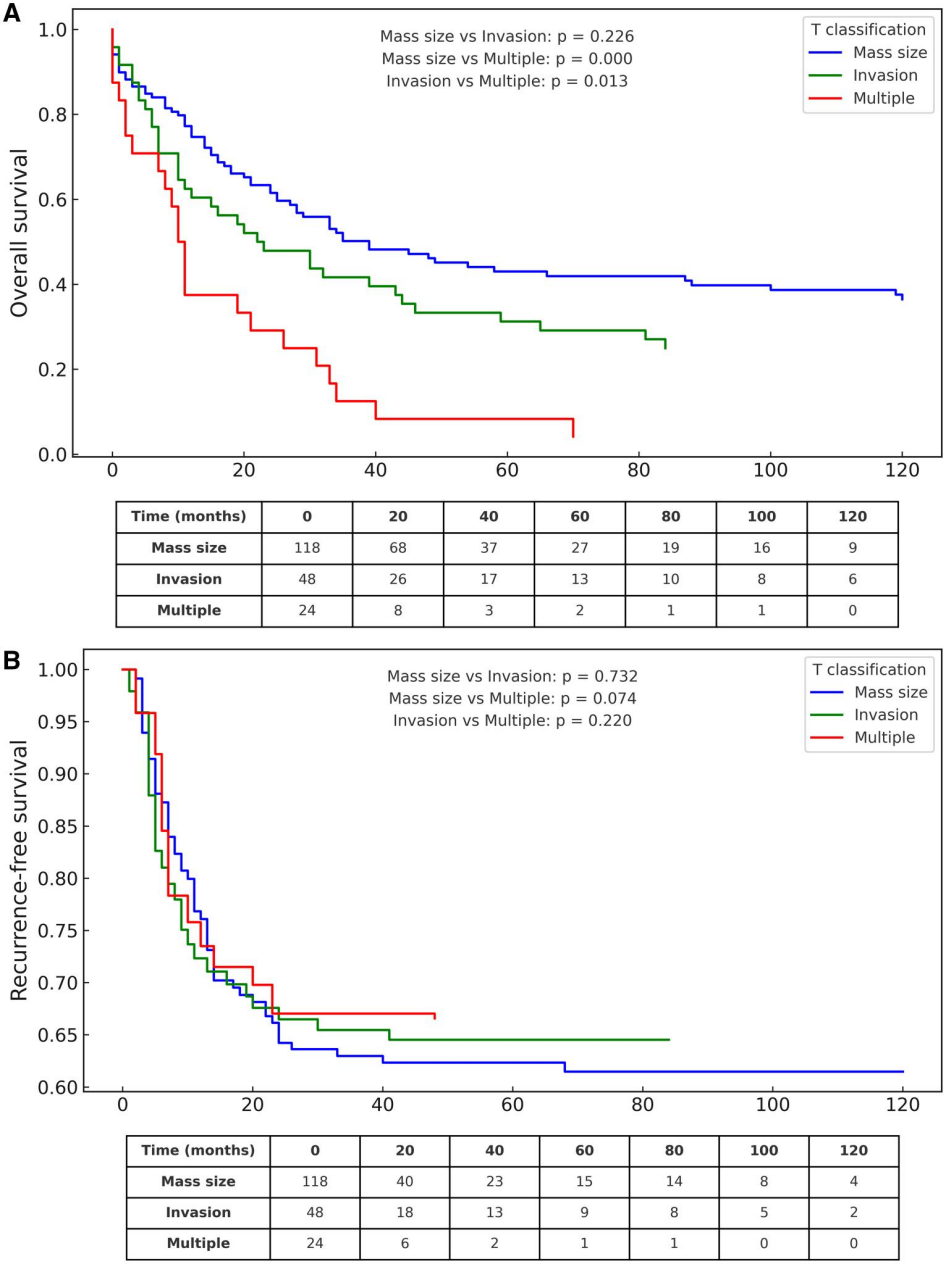
Overall Survival (OS) and Recurrence-Free Survival (RFS) According to T4 Subclassification. (A) OS curves (Kaplan-Meier) by T4 subgroups: size >7 cm (*n* = 119), invasion of adjacent structures (*n* = 48), and multiple T4 criteria (*n* = 24). Survival over the entire follow-up period differences were assessed with the log-rank test. (B) RFS curves (Aalen-Johansen) for the same T4 subgroups, with death treated as a competing risk. Patients at risk at specific time points are shown below the curves.

The 1-, 2- and 5-year OS rates for the entire cohort were 69.6%, 52.0% and 34.1%, respectively. Survival over the entire follow-up period was significantly different in T4 subgroups. OS was worse in the multiple T4 group (median OS: 10 months) compared to the invasion (median OS: 22 months) and mass size (median OS: 34 months) groups (**Figure 1A**). Median differences in OS and RFS among T4 subgroups are presented to illustrate the magnitude of survival variation.

### RFS, according to T4 subgroups

The 5-year RFS for the entire cohort was 36.7%. Recurrence over the entire follow-up period was not significantly different in T4 subgroups. Although recurrence occurred earlier in some patients from the multiple T4 group, overall differences among the 3 subgroups did not reach statistical significance (**Figure 1B**). While visual inspection of the curves suggests potential separation over time, these trends were not statistically confirmed and should be interpreted cautiously.

### Nodal status and survival

**Figure 2.**
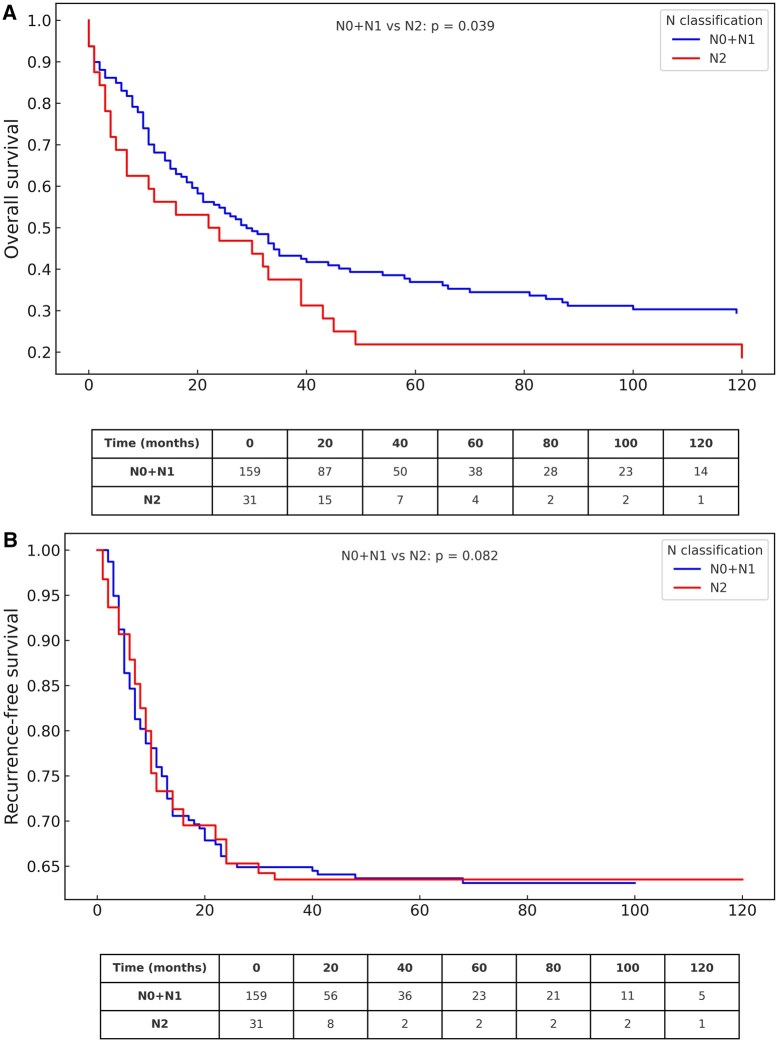
Survival Analysis According to Nodal Status. (A) Overall survival curves (Kaplan-Meier) for patients with N0-1 (*n* = 158) vs N2 (*n* = 32). Survival over the entire follow-up period differences were evaluated with the log-rank test. (B) Recurrence-free survival curves (Aalen-Johansen) by Nodal Status (N0-1 vs N2), with death as a competing risk. Patients at risk at specific time points are shown below the curves.

Patients with N2 disease had worse OS than those with N0-1 (median 22 vs 29 months, *P* = .039, **Figure 2A**). RFS differences were not statistically significant (*P* = .082, **Figure 2B**), though curves indicated a more aggressive course in N2-positive patients that may still be clinically relevant. The negative prognostic effect of N2 was evident in the invasion group (median 7 vs 44 months, *P* = .001), but minimal in the size group (median 45 vs 33 months, *P* = .896), and modest in the multiple group (median 7 vs 11 months, *P* = .209). Overall, nodal impact appeared context-dependent and should be interpreted alongside T4 features. In total, 32 patients had pathological N2 disease, including six with unexpected multi-station (pN2b).

### AChT and recurrence outcomes

**Figure 3.**
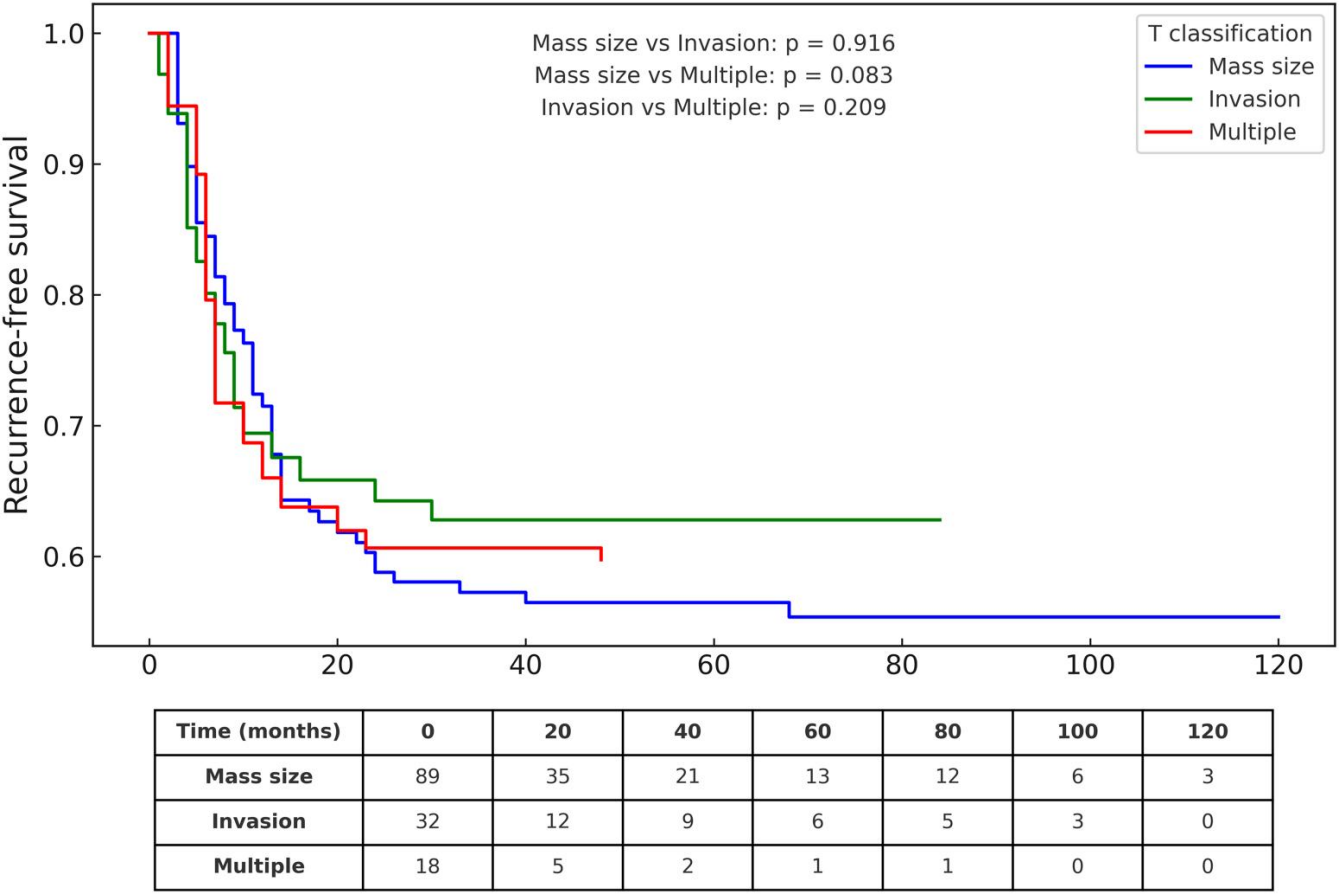
RFS According to Adjuvant Chemotherapy (AChT) Status Across T4 Subgroups. Recurrence-free survival curves (Aalen-Johansen) for patients who received AChT (*n* = 139) vs those who did not (*n* = 52), by T4 subgroup (size, invasion, multiple). Patients at risk are shown below the curves.

Among patients who received AChT (*n* = 139), the mean RFS was highest in the mass size group (71.6 months), moderate in the invasion group (55.4 months), and lowest in the multiple T4 group (31.8 months). Although differences were not statistically significant (**Figure 3**), the decline in the multiple group suggests more aggressive biology despite adjuvant therapy, indicating limited efficacy in this subgroup.

### Multivariable Cox regression analysis

Multivariable analysis (**Table 4**) showed AChT as a favourable factor (hazard ratio [HR]: 0.511, *P* = .001), while pathological N2 independently predicted poor survival (HR: 1.750, *P* = .012), consistent with its role as an established negative prognostic marker. Multiple T4 features emerged as the strongest adverse predictor (HR: 2.590, *P* < .001), suggesting overlapping criteria confer a compounded biological disadvantage. Other demographic, surgical, and pathological factors were not significant. The multivariable Cox model included clinically relevant variables based on prior evidence; however, given the limited number of events, the potential for mild model overfitting cannot be excluded.

**Table 4.** Univariable and Multivariable Cox Regression Analysis of Prognostic Factors for OS

	Univariable analysis		Multivariable analysis	
	HR (95% CI)	*P*-value	HR (95% CI)	*P*-value
Age	1.029 (1.007-1.051)	**.009**	1.017 (0.995-1.039)	.127
Gender				
Female	1			
Male	0.551 (0.279-1.087)	.085		
Operation type				
Pneumonectomy	1			
Lobectomy	0.909 (0.641-1.291)	.595		
Histopathology				
Squamous cell carcinoma	1	.757		
Adenocarcinoma	0.881 (0.568-1.366)	.571		
Others	1.078 (0.686-1.694)	.744		
Adjuvant chemotherapy				
No	1		1	
Yes	0.532 (0.363-0.778)	.001	0.511 (0.344-0.758)	.001
Adjuvant radiotherapy				
No	1			
Yes	1.121 (0.718-1.750)	.615		
Pathologic lymph node				
N0-1	1		1	
N2	1.563 (1.015-2.406)	.043	1.750 (1.129-2.713)	.012
T subgroups				
Mass size	1	.001	1	.001
Invasion	1.280 (0.856-1.915)	.229	1.114 (0.741-1.676)	.604
Multiple	2.552 (1.586-4.106)	.001	2.590 (1.578-4.250)	.001
Complication				
No	1			
Yes	1.332 (0.896-1.979)	.157		
Tumour location				
Main bronchus	1	1.000		
Right	1.006 (0.629-1.610)	.980		
Left	1.005 (0.637-1.585)	.983		

Hazard ratios (HR) with 95% CIs are shown. Variables included age, sex, histology, T4 subgroup, nodal status, reoperation, and adjuvant chemotherapy (AchT) and adjuvant radiotherapy. In the final model, multiple T4 features and N2 disease were independent predictors of worse overall survival (OS), while AChT was protective. During follow-up, 129 death events occurred, which is an adequate number relative to the included covariates and supports model stability.

## DISCUSSION

Our analysis of surgically treated T4 NSCLC patients, staged per the 9th TNM edition, showed a 5-year OS of 34.1%, consistent with prior reports.^9^^,^^10^ These findings confirm surgery as a viable option in selected T4 cases, as also reported by Kaba et al. (2-year OS 61.4% after upfront surgery and adjuvant therapy).^11^

The heterogeneity of T4 disease, reflected in the TNM classification, contributes to variable outcomes.^2^^,^^12^ In our series, survival was best in size-only, lower in invasion-only, and poorest in multiple T4 cases, suggesting greater biological aggressiveness. Multiple adverse features may represent both increased tumour burden and distinct tumour biology. The lack of molecular data limited confirmation, highlighting the need for future studies integrating clinical, pathological, and molecular factors.

Prior studies on T4 subgroups have shown inconsistent results, reflecting classification heterogeneity. Watanabe et al. found no survival differences by invasion type, while Li et al. reported better invasion outcomes than size-based T4.^13^^,^^14^ Aksoy et al. found no subgroup differences,^15^ and Erdogu et al. noted similar survival for tumours >10 cm and 7-9.9 cm.^16^ Wang et al. highlighted the prognostic impact of both lesion size and distribution.^17^ The multiple T4 group in our cohort showed significantly poorer survival (HR = 2.59; *P* < .001). RFS, analysed with the Aalen-Johansen competing risk method, confirmed earlier and more frequent recurrence in this group, despite limited statistical significance. Our findings underscore the need for greater caution when considering surgery, as multiple T4 features may indicate biologically aggressive disease.

Lymph node status is a key prognostic factor. In our study, N2 patients had significantly shorter OS than N0-1 (22 vs 29 months, *P* = .039), and N2 was an independent predictor of poor prognosis (HR = 1.75; *P* = .012). Although RFS was not statistically significant, curves diverged, suggesting a more aggressive course in N2-positive cases. Subgroup analysis showed a strong adverse effect of N2 in the invasion group (7 vs 44 months; *P* = .001), while in the multiple group its impact was not significant—likely due to the small sample size and the overwhelming effect of the T4 characteristics—but a trend towards worse outcomes was observed. Among 32 N2 patients (26 N2a, 6 N2b), all N2b cases were identified postoperatively, highlighting challenges in staging. These results align with reports showing N2 heterogeneity: single-station N2 may yield meaningful survival, whereas multi-station N2 conveys distinctly worse outcomes.^18^

Asmara et al. confirmed the clinical value of distinguishing N2a from N2b,^19^ and Wen et al. reported superior survival with surgery versus radiotherapy in selected T4-N2 patients.^20^ Consistent with these findings, our study reinforces that only well-selected patients with limited N2 involvement may benefit from surgery, whereas unexpected multi-station N2 is associated with an inferior prognosis.

AChT significantly improved survival in our series and emerged as an independent favourable factor (HR = 0.511; *P* = .001). These findings are consistent with Topaloglu et al. and Song et al., who demonstrated survival benefits from AChT following extended resections or in large T4N0 tumours.^21^^,^^22^ However, adherence to chemotherapy was suboptimal: many of the 52 patients who did not receive or could not complete planned adjuvant treatment had postoperative morbidity or impaired performance status. This underscores that AChT benefit depends on recovery and patient selection rather than uniform application.^23^

The prognostic role of ARdT remains debated. Wang et al. reported potential adverse effects in tumours >5 cm with N0 status.^24^ Our cohort applied ARdT exclusively to N2 patients, completely overlapping treatment and nodal status. In multivariable analysis, ARdT was not an independent factor, suggesting that poor outcomes were driven mainly by nodal disease. Thus, the use of ARdT for incidentally detected N2 should be individualized based on margin status, nodal burden, and patient fitness. Only a small minority (*n* = 8) received adjuvant immunotherapy, mainly in the later years, and reimbursement restrictions prevented broader use, precluding meaningful analysis.

The T4 category includes large tumours and those invading adjacent structures. Our data show that extended resections of major vessels, airway, or organs such as vertebra or diaphragm can be safely performed in experienced centres, provided R0 is achieved.^25^^,^^26^ Surgery also preserved survival in selected superior vena cava-invaded cases, with graft reconstruction further improving outcomes.^27–29^ In left atrial invasion, survival was favourable with R0 and neoadjuvant therapy^30^^,^^31^; 2 R2 cases were excluded. Even vertebral invasion can yield benefit: Novellis et al. reported 20% 5-year OS, while Oka et al. achieved 71.4% with high morbidity.^32^^,^^33^ Our findings were comparable. Carinal invasion also showed survival consistent with prior reports.^34^^,^^35^ Overall, prognosis varies by invasion site, underscoring the need for site-specific analysis. Incorporating invasion patterns and nodal subclassification in the 9th TNM supports a more tailored surgical approach.

Therefore, a T4 tumour classified by size >7 cm alone appears to have a more favourable biology and is a stronger candidate for aggressive surgery than a tumour exhibiting invasion or, especially, multiple T4 features.

### Strengths and limitations

The main strength of this study is its large, uniformly managed cohort of T4 NSCLC cases staged per the 9th TNM edition. Systematic lymph node dissection, R0 resections, and standardized techniques enhance reliability. Subgroup analysis and multivariable Cox regression further support robustness.

Limitations include the retrospective single-centre design and the long inclusion period (2006-2024), which may reflect changes in surgical and oncologic practices. Subgroup sizes, particularly invasion and multiple T4, were small, reducing statistical power. Molecular data were unavailable, and only eight patients received adjuvant immunotherapy, precluding meaningful analysis. The male predominance (94.8%), reflecting regional smoking patterns, may limit generalizability. Some cases initially staged as single-station N2 were later reclassified as multi-station, highlighting staging challenges.

Although the multivariable model adjusted for key clinical factors, the event-to-covariate ratio may have caused mild overfitting, acknowledged as a limitation. RMST analysis was infeasible due to incomplete follow-up; thus, survival comparisons relied on log-rank testing interpreted over the follow-up period.

## CONCLUSION

This study highlights key prognostic factors in surgically treated T4 NSCLC. Patients with N2 disease or multiple T4 criteria had the worst outcomes, whereas AChT improved survival. Multiple T4 involvement indicated particularly poor prognosis and should be considered a critical determinant in treatment planning. The 9th TNM edition provides a valuable framework for future stratification.

## Data Availability

The data presented in this study are available in the institutional archive of Gaziantep University upon request.
